# Synergistic effect of human Bone Morphogenic Protein-2 and Mesenchymal Stromal Cells on chronic wounds through hypoxia-inducible factor-1 α induction

**DOI:** 10.1038/s41598-017-04496-w

**Published:** 2017-06-27

**Authors:** Sabine François, Véronique Eder, Karim Belmokhtar, Marie-Christine Machet, Luc Douay, Norbert-Claude Gorin, Marc Benderitter, Alain Chapel

**Affiliations:** 1 Laboratory of Research on Irradiated Healthy Tissue Regeneration (LR2I), Institute for Radiological Protection and Nuclear Safety (IRSN), F-92260 Fontenay-aux-Roses, France; 2 Proliferation and Differentiation of Stem Cells, Centre de Recherche Saint-Antoine (CRSA), UMR_S938, Faculté de médecine Pierre et Marie Curie, France Institut National de la Santé et de la Recherche Médicale (INSERM) U938, 27 rue de Chaligny, 75012, Paris, Paris, France; 3 LAB.P.ART.-EA3852 Faculty of Medicine, University of Tours, 2 bis boulevard Tonnellé, 37000 Tours, France

## Abstract

Chronic skin ulcers and burns require advanced treatments. Mesenchymal Stromal Cells (MSCs) are effective in treating these pathologies. Bone Morphogenic Protein-2 (BMP-2) is known to enhance angiogenesis. We investigated whether recombinant human hBMP-2 potentiates the effect of MSCs on wound healing. Severe ulceration was induced in rats by irradiation and treated by co-infusion of MSCs with hBMP-2 into the ulcerated area which accelerated wound healing. Potentiation of the effect of MSCs by hBMP-2 on endothelial repair improved skin healing. HBMP-2 and MSCs synergistically, in a supra additive or enhanced manner, renewed tissue structures, resulting in normalization of the epidermis, hair follicles, sebaceous glands, collagen fibre density, and blood vessels. Co-localization of MSCs with CD31 + cells suggests recruitment of endothelial cells at the site of injection. HBMP-2 and MSCs enhanced angiogenesis and induced micro-vessel formation in the dermis where hair follicles were regenerated. HBMP-2 acts by causing hypoxia-inducible factor-1 α (HIF-1α) expression which impacts endothelial tube formation and skin repair. This effect is abolished by siRNA. These results propose that new strategies adding cytokines to MSCs should be evaluated for treating radiation-induced dermatitis, burns, and chronic ulcers in humans.

## Introduction

Chronic wounds significantly impair the quality of life of millions of people and remain a major challenge in modern medicine. The treatment of burns and chronic skin ulcers still requires more effective therapy^1–4^. Cutaneous irradiation may cause chronic, torpid long lasting ulcerations^5,6^. Microvascular lesions with endothelium dysfunction play a key role in their pathogenesis^7–9^. In the last two decades, extensive laboratory, preclinical and clinical studies of stem cells have provided new perspectives for the management of chronic non-healing wounds^10^. Local and/or systemic administration of progenitor/stem cells, mesenchymal stromal cells (MSCs), in particular harvested from bone marrow (BM) or adipose tissue, have been tested in patients suffering from many chronic diseases^11–14^. MSCs have the potential to improve limb ischemia, systemic scleroderma, ulcerative wounds in diabetes, severe skin damage, and thermal and radiation burns^15–19^. They have also been used for aesthetic and reconstructive surgery^16^. Recent case reports have highlighted the favourable therapeutic results in victims of radiation accidents with severe cutaneous ulcerations, by the combination of local MSC administration and surgery, drawing attention to the angiogenic effect of MSC infusion^20^.

MSCs have been used in the field but the results have remained somewhat uncertain. The scientific question we are addressing is whether we can improve the efficacy of stem cell therapy for ulcerative damage, with co-administration of selected cytokines. The treatment of chronic wounds remains a major challenge in modern medicine where guidelines for clinical use are required to increase the effects of MSCs. Several parameters must be optimized such as route of delivery, doses and frequency of cell administration. Furthermore, distribution of MSCs in tissue and molecular mechanisms of chemotaxism remain unclear. Finally, the use of drug treatment in support of MSC infusion should be addressed to increase wound healing efficacy^21^.

Bone morphogenic proteins are members of the TGF beta family^22,23^. HBMP-2 is a 396 aa glycosylated polypeptide. The mature region has seven cysteines and one N-linked glycosylation site. With a predicted mass of 14 kDa, the mature segment is actually 18 kDa and is assumed to be glycosylated. The functional form of the molecule consists of two disulfide-linked mature chains. There is 100% identity in the mature region of human, mouse, and rat protein^24^.

HBMP-2 is used in clinical practice to enhance angiogenesis and osteogenesis surrounding hip end prostheses with repair defects. *In vitro* experiments have shown that hBMP-2 plays an important role in the chemotaxis of microvascular endothelial cells^25^. HBMP-2 induces chemotaxis, mesenchymal cell proliferation and differentiation, and angiogenesis. HBMP-2 controls extracellular matrix synthesis and plays an important role in the healing process. HBMP-2 stimulates the synthesis and angiogenic secretion of growth factors and directly-activates endothelial cells for angiogenesis (in part via the HIF-1α pathway), and regulates the healing process. HBMP-2 also improves healing by promoting dermal and epidermal growth leading to keratinized and thickened skin^26^. HBMP-2 has been involved in the TNF-α-induced EMT in human skin^27^. Skin wound healing is a complex oxygen-dependent process. Hypoxia-inducible factor (HIF) serves as a crucial oxygen sensor and plays an important role during reepithelialisation and increased expression of hBMP-2. HIF-1 plays an important role in skin homeostasis during ageing and loss of HIF-1 leads to a delay in wound healing^28–30^.

In this study we investigated whether hBMP-2 potentiates the effect of MSCs to minimize ulceration and improve wound healing during radio dermatitis. We hypothesized that a combination of hBMP-2 and MSCs enhances angiogenesis and microvascular development, improving skin repair through a HIF-1-dependant mechanism. We found that the addition of hBMP-2 to MSCs regenerated tissue structures completely, resulting in normalization of the epidermis, hair follicles, sebaceous glands, collagen fibre density, and blood vessels. *In vivo*, local infusion of MSCs + hBMP-2 into radiation-induced dermatitis lesions revealed the co-localization of MSCs with CD31 + cells, suggesting recruitment of endothelial cells at the site of injection. HBMP-2 induced HIF-1 expression in MSCs, which could impact endothelial tube formation. Expression of HIF-1α in the dermis was significantly increased with MSC plus hBMP-2 treatment. This observation reveals a tissue-specific action from hBMP-2 and HIF-1α in the dermis. Silencing of the HIF-1α gene inhibited the effect of hBMP-2 on endothelial tube formation.

## Results

### Clinical and Histological Characterization of radiation-induced dermatitis

**Figure 1:**
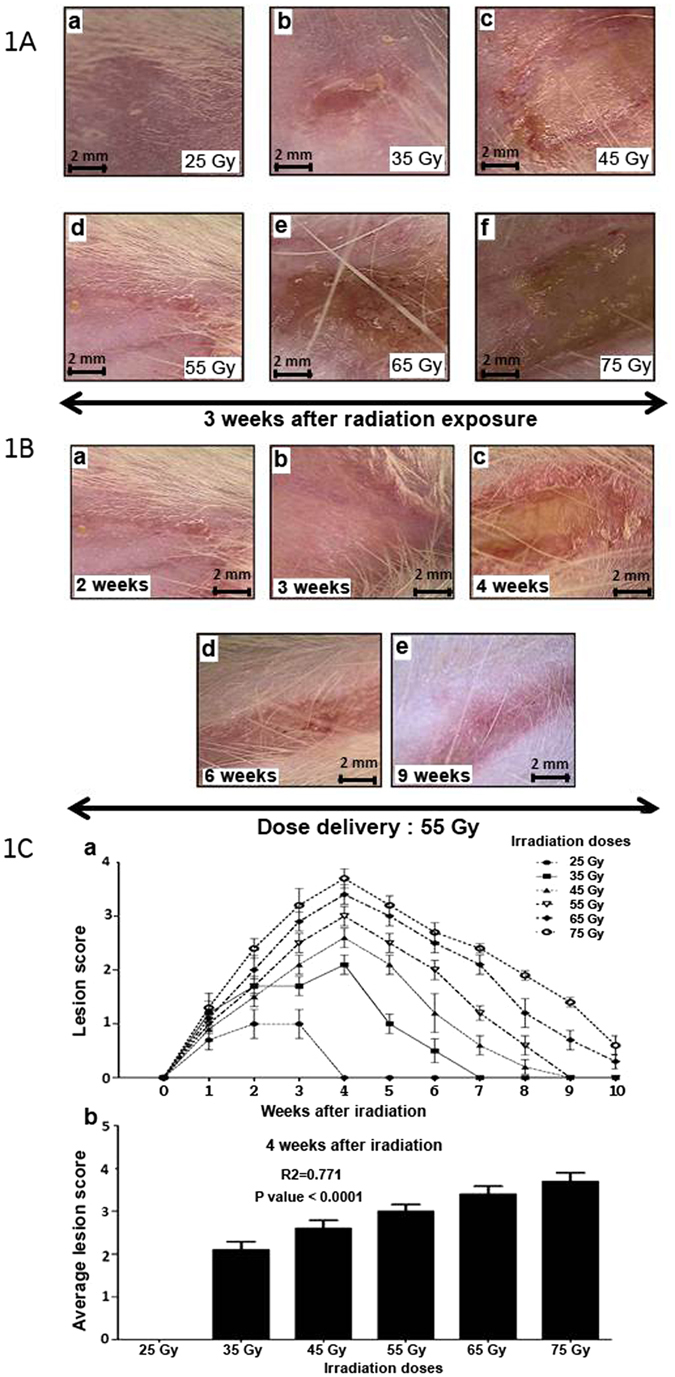
Skin lesions based on radiation dose in a radiation-induced dermatitis model. (**A**) Photographs of radiation-induced lesions 3 weeks post-exposure: (a) 25 Gy, (b) 35 Gy and (c) 45 Gy, (d) 55 Gy, (e) 65 Gy, (f) 75 Gy. (**1B**) Macroscopic time-lapse observation of skin lesions following 55 Gy exposure. (**1C**) (a) Severity of lesions. (b) Average lesion score 4 weeks after irradiation. 14 groups (n = 10 rats in each group). The first group consisted of un-irradiated rats. Five groups were irradiated at 25, 35, 45, 55, 65, and 75 Gray (Gy). Each group of animals comprised 10 Sprague Dawley rats. Comparison by ANOVA followed by the Mann-Whitney or Dunnett’s test for group pair-wise comparisons. Data represent mean ± SEM, ns: not significant, *p < 0.05, **p < 0.01, ***p < 0.001.

In an effort to develop a severe radiation burn radiation-induced dermatitis model, the right posterior limbs of rats were irradiated locally. After removing the hair from the right posterior leg, the area was exposed to a single dose of ionizing radiation ranging from 25 to 75 Gray. The lesion was limited to the exposed internal face of the leg. Hair re-growth was not observed in this area until 4 weeks after irradiation. Figure 1A shows the damage to the exposed skin for each dose 3 weeks after irradiation. Acute radiation-induced dermatitis lesions were classified based on the National Cancer Institute Common Terminology Criteria For Adverse Events version 3^31^. Exposure of 25 Gray (Gy) resulted in grade 1 radiation-induced dermatitis with dry desquamation (Fig. 1Aa), 35 Gy and 45 Gy induced grade 2 and 3 radiation-induced dermatitis corresponding to moist desquamation (Fig. 1Ab,c), 55 Gy induced ulceration equivalent to grade 4 radiation-induced dermatitis (Fig. 1Ad and Ba–e), and 65 Gy and 75 Gy provoked necrosis correlating to grade 4 radiation-induced dermatitis with increased severity (Fig. 1Ca,b). The highest degree of lesion severity was reached 4 weeks post-irradiation (Fig. 1Bc); therefore, we studied the dose effect at this time point. The average score for the cutaneous and subcutaneous lesions at 4 weeks was 2.1 to 3.7 from 35 Gy and 75 Gy, respectively (Fig. 1Ba,b). We demonstrated a significant correlation (R^2^ = 0.7741, *p* < 0.0001) between average lesion score and radiation dose delivered (Fig. 1Ca,b). This observation indicates that lesion surface area and severity are dose-dependent.

### MSC Effect on Ulceration and Wound Healing

**Figure 2:**
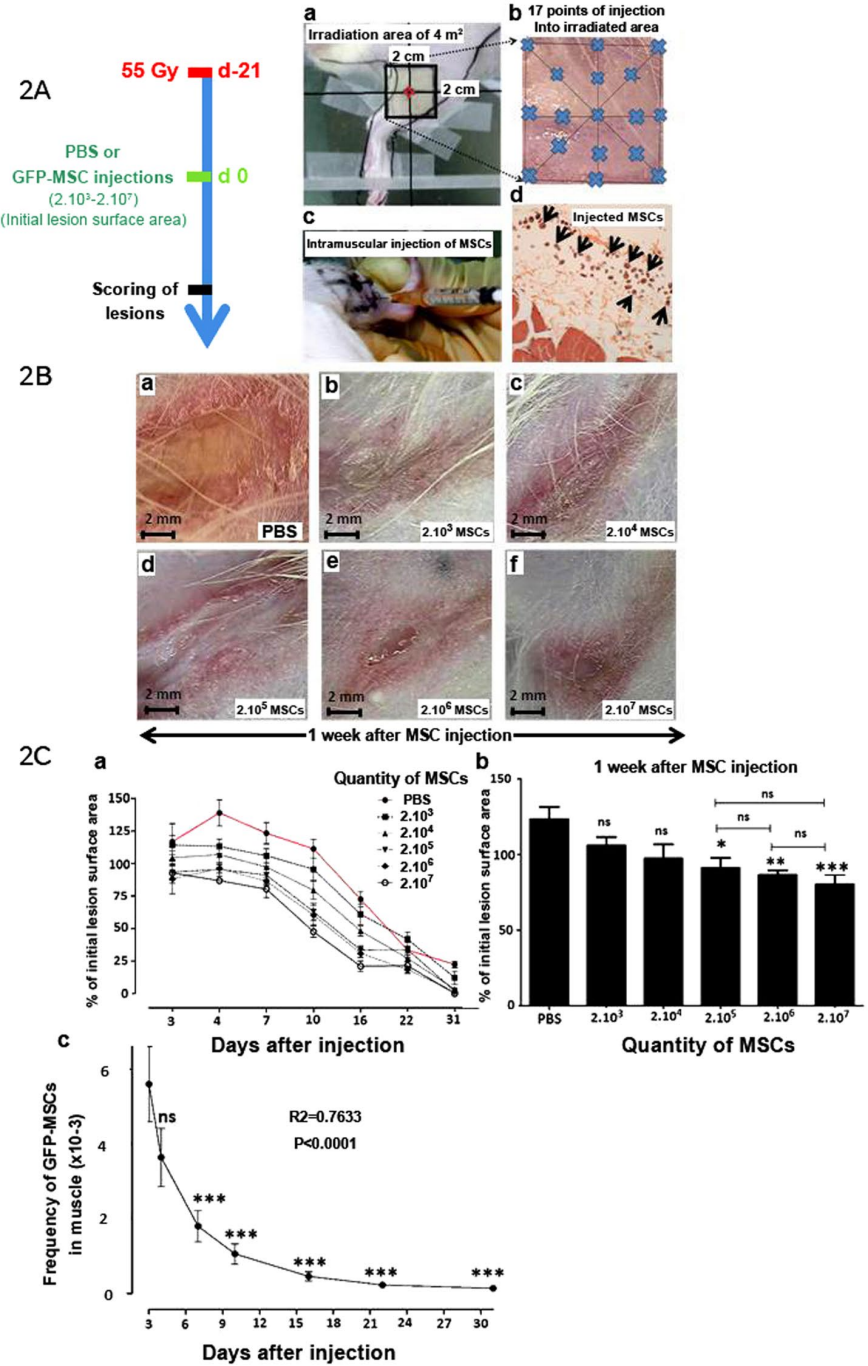
Effect of MSCs on Ulceration and Wound Healing. (**A**) Protocol and local MSC injection procedure: Three groups of irradiated animals were irradiated at 55 Gy and received PBS (PBS), MSCs (MSCs) and hBMP-2 (MSCs + hBMP-2) infusion three weeks later. (a) Picture of the specific irradiation area, (b) illustration of intramuscular injection sites (blue star), (c) MSC intramuscular injection, and (d) localization of injected cells by histological HES staining (black arrows). (**B**) Change in irradiated skin lesions 1 week after treatment with (a) PBS alone or (b–f) various quantities of MSCs ranging from 2 × 10^3^ to 2 × 10^7^ cells. (**C**) (a) Relative lesion surface area progression. (b) Relative lesion surface area in comparison with the PBS control infusion. (c) Quantification of GFP-MSCs in muscle using quantitative GFP PCR detection. Each group of animals comprised 10 Sprague Dawley rats. Comparison by ANOVA followed by the Mann-Whitney or Dunnett’s test for group pair-wise comparisons. Data represent mean ± SEM, ns: not significant, *p < 0.05, **p < 0.01, ***p < 0.001.

In a protocol similar to that used in patient treatment, MSCs were injected around and into the lesion in the skin and muscle (Fig. 2A). For patients, local administration consisted in repeated injections of more than 150 million MSCs which were injected in a circle around the lesion at cutaneous and muscular level^20^. MSCs were isolated from the BM of eGFP transgenic rats in order to track the cells after engraftment in recipient rats, which were GFP-negative. We chose a 55 Gy irradiation dose as it induces a severe lesion with ulceration and requires an average healing period of 5 weeks. MSCs were implanted 3 weeks after irradiation (see Fig. 2A) by 17 intramuscular infusions into the ulcerated area and periphery (Fig. 2Ab, blue stars), from 5 mm deep into the muscle up to the skin surface (Fig. 2Aa–c). Histological sections using HES (Hematoxylin, Eosin, Saffron) staining (Fig. 2Ad) confirmed the MSCs (black arrows) to be evenly distributed around the muscle and skin (visualized by round cells). Macroscopic observations of the radiation-induced dermatitis 1 week post-transplantation revealed apparently smaller and less severe lesions with an increasing number of MSCs, ranging from 2 × 10^3^ to 2 × 10^7^ (Fig. 2Bb–f), compared to the PBS control (Fig. 2Ba). As shown in Fig. 2Ca, local infusion of MSCs significantly decreased the lesion surface area as early as day 4 for all quantities of MSCs tested. MSC infusion improved lesion repair up to 31 days post-infusion, except for 2 × 10^3^ MSCs, which had greater variance than any other treatment condition. To determine the optimum number of MSCs to infuse, the benefit with regards to decreased lesion surface area was analyzed 7 days post-transplantation (Fig. 2Ca,b), when the effect of transplantation is at a minimum and skin damage is at the highest levels. A significant decrease in lesion surface area required a minimum of 2 × 10^5^ MSCs. Infusion of additional MSCs, i.e. 2 × 10^6^ and 2 × 10^7^, did not further improve lesion repair.

As seen in Fig. 2Cc, GFP-MSCs were detected by qPCR using GFP-specific primers up to 31 days post-transplantation. GFP-MSCs decreased rapidly the first 2 weeks post-infusion following one-phase exponential decay and remained stable and for at least 31 days, reaching a plateau on day 16 (R^2^ = 0.7633, *p* < 0.0001). Finally, MSCs improved repair from 4 to 7 days post-infusion, and a minimum of 2 × 10^5^ MSCs was required.

### Local co-injection of hBMP-2 and MSCs attenuated the progression of radiation-induced dermatitis and accelerated the healing process

**Figure 3:**
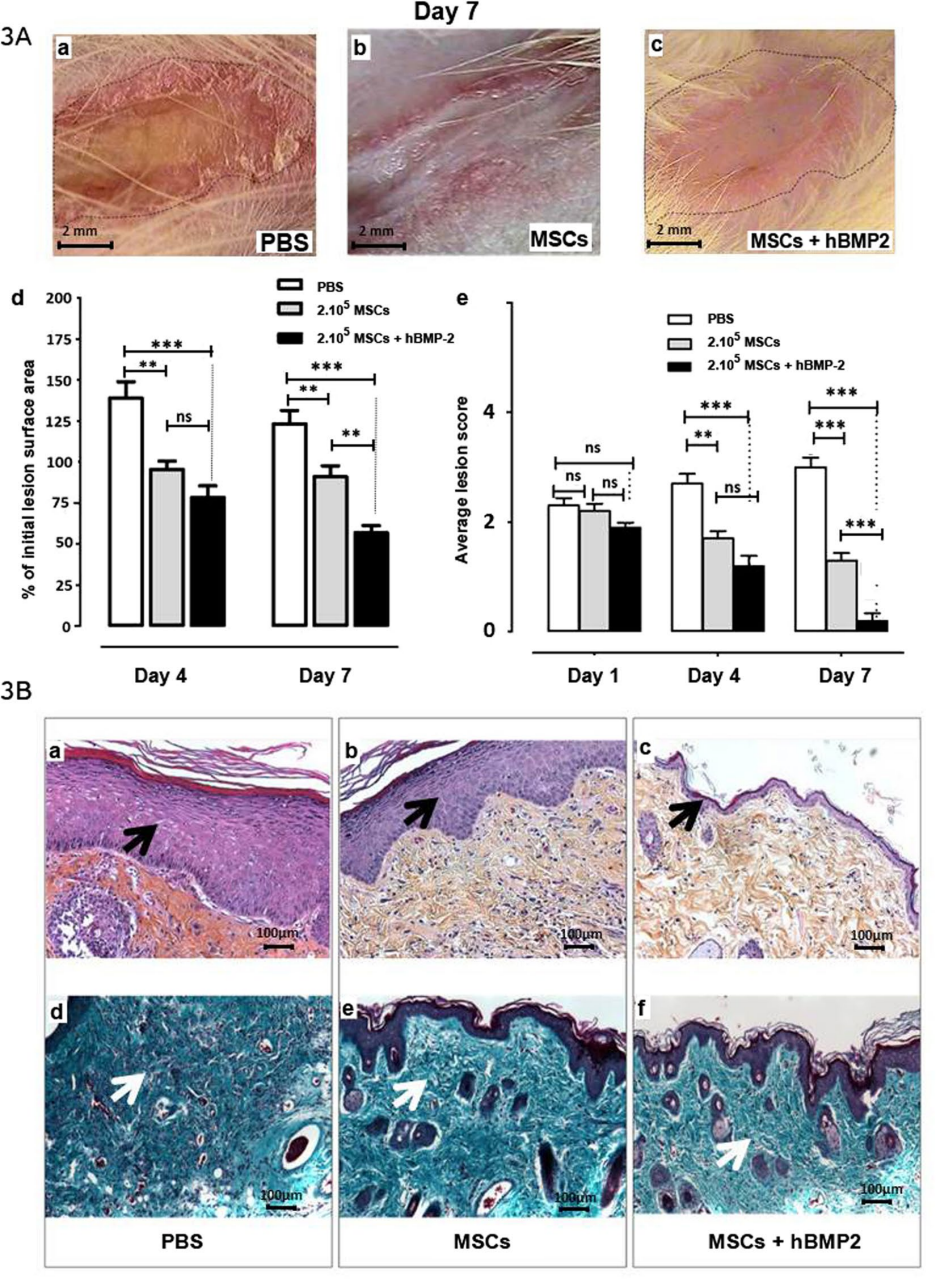
Effect of hBMP-2 addition to MSC therapy on radiation-induced dermatitis repair. (**A**) Photographs of radiation-induced lesions 7 days after injection. Three groups of animals were irradiated at 55 Gy and received PBS (PBS), MSCs (MSCs), or MSCs and hBMP-2 (MSCs + hBMP-2) infusion three weeks later. (a) PBS treatment, (b) infusion of 2 × 10^5^ MSCs, and (c) co-injection of 2 × 10^5^ MSCs and hBMP-2. (d) % of initial lesion surface area and (e) average lesion scores. PBS (white bars), MSCs (grey bars), or MSCs + hBMP-2 (black bars). (**B**) One week after treatment, HES staining (a–c) (a) PBS control infusion; (b) local infusion of 2 × 10^5^ MSCs; and (c) hBMP-2 co-infusion with MSCs. (d–f) Masson’s Trichrome staining of: (d) PBS control infusion; (e) local infusion of 2 × 10^5^ MSCs; and (f) hBMP-2 co-infusion with MSCs. Each group of animals comprised 10 Sprague Dawley rats. Comparison by ANOVA followed by the Mann-Whitney or Dunnett’s test for group pair-wise comparisons. Data represent mean ± SEM, ns: not significant, *p < 0.05, **p < 0.01, ***p < 0.001.

We evaluated the effect of co-injecting hBMP-2 and 2 × 10^5^ MSCs (MSCs + hBMP-2) on skin lesion repair (Fig. 3) at 4 and 7 days post-infusion. PBS-treated control rats developed full ulceration of the irradiated area (Fig. 3Aa), whereas animals treated with MSCs (Fig. 3Ab) or MSCs + hBMP-2 (Fig. 3Ac) displayed partially healed lesions in the moist and dry desquamation phase, respectively. Co-treatment of radiation-induced dermatitis with MSCs + hBMP-2 improved tissue healing compared to MSC infusion alone, based on a reduction in both lesion surface area (Fig. 3Ad) and average score (Fig. 3Ae). Linear trend modelling of average lesion score depicted a decrease in radiation-induced dermatitis severity following MSC treatment without (R^2^ = 0.6903, *p* = 0.0002) and with hBMP-2 (R^2^ = 0.8401, *p* < 0.0001) compared to the PBS control (R^2^ = 0.4324, *p* = 0.0104). HBMP-2 co-infusion with MSCs hastened lesion healing 1.9-fold (slope = −0.45 and −0.85 for MSCs and MSCs + hBMP-2, respectively). Thus, we demonstrated that MSCs + hBMP-2 not only stops the progression of damage to the irradiated tissues, but also accelerates the repair process.

**Figure 4:**
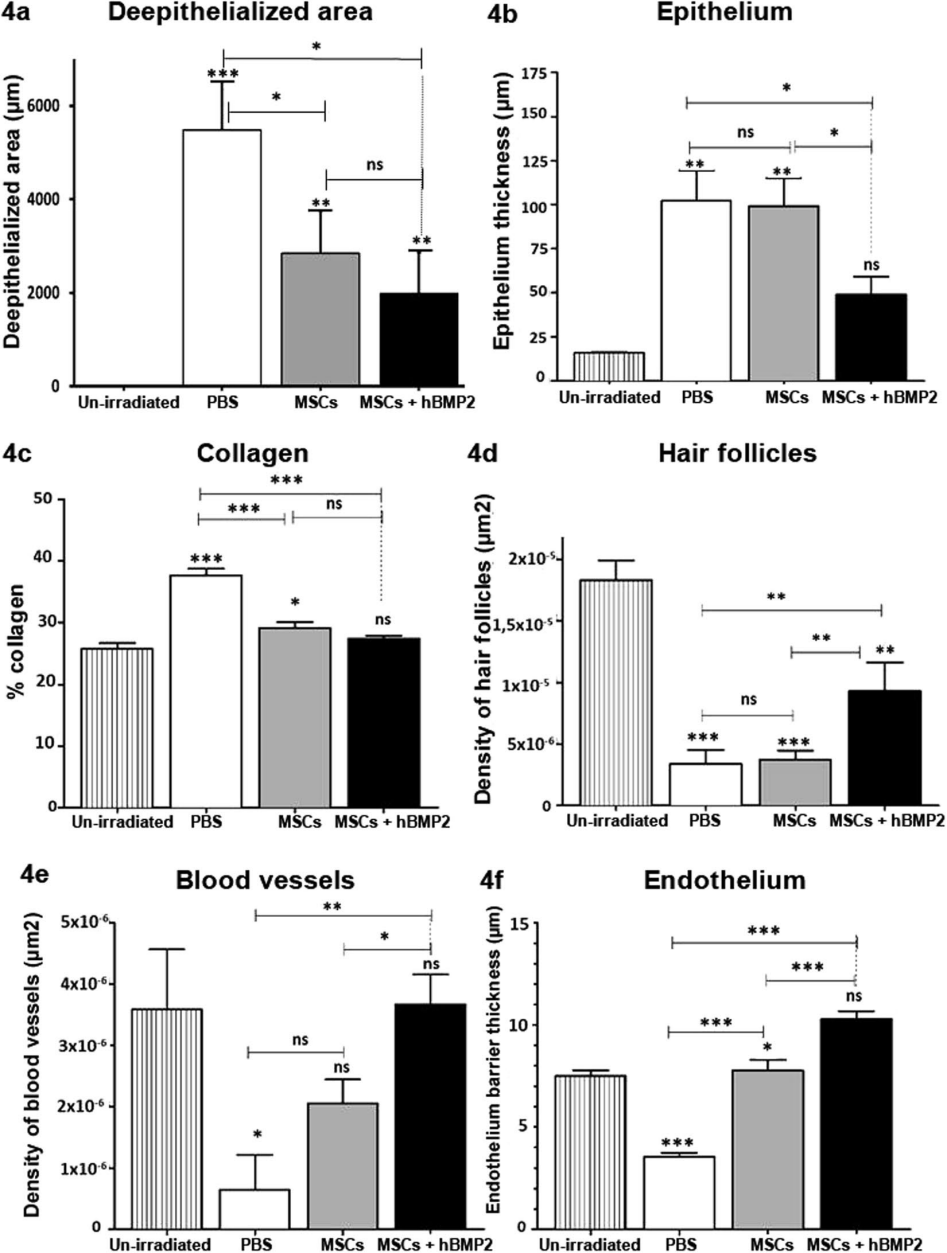
Quantification of skin tissue structures in the epidermis and dermis one week post-treatment. Three groups irradiated were irradiated at 55 Gy and received PBS (PBS), MSCs (MSCs), or MSCs and hBMP-2 (MSCs + hBMP-2) infusion three weeks later. (**a**) deepithelialized area, (**b**) epithelium thickness, (**c**) collagen density, (**d**) hair follicle density, (**e**) blood vessel density, and (**f**) endothelial barrier thickness were analyzed using Histolab software. Each group of animals comprised 10Sprague Dawley rats. Comparison by ANOVA followed by the Mann-Whitney or Dunnett’s test for group pair-wise comparisons. Data represent mean ± SEM, ns: not significant, *p < 0.05, *p < 0.01, ***p < 0.001.

Microscopic histopathological examination by HES and Masson Trichrome staining 1 week post-treatment is shown in Fig. 3B. In the dermis, we observed a reduction in the number of blood vessels, hair follicles, and sebaceous glands, and an increase in collagen fibre density in PBS-treated rats (Fig. 3Ba,d). Figure 3Bb,e depict typical improvement in skin repair following local infusion of 2 × 10^5^ MSCs, resulting in reduced dermal hypertrophy and accelerated skin restructuring as shown by the presence of blood vessels and decreased collagen fibre density. The addition of hBMP-2 to the local infusion of MSCs fully regenerated tissue structures (Fig. 3Bc,f). To evaluate the impact of hBMP-2 co-infusion with MSCs on the skin repair process, we quantified the variation in epidermal hypertrophy (Fig. 4a,b), collagen (Fig. 4c), and hair follicle density (Fig. 4d), and the number and thickness of blood vessels (Fig. 4e,f) in the entire irradiated area. First, we determined that the de-epithelialized area (area without epidermis) was reduced from 5483 ± 1036 µm (mean ± SEM) measured with the control PBS treatment to 2839 ± 916 µm following MSC treatment alone, and 1980 ± 895 µm following MSCs + hBMP-2 co-infusion. The MSCs + hBMP-2 co-treatment significantly reduced (one-way ANOVA with Dunnett’s MCT, *p* < 0.05) the de-epithelialized surface area 2.8-fold compared to the control PBS treatment (Fig. 4a).

MSCs + hBMP-2 treatment significantly decreased collagen density to a level comparable to the un-irradiated control (Fig. 4c). Co-treatment of radiation-induced dermatitis with MSCs and hBMP-2 is necessary to significantly increase hair follicle density in the dermis (Fig. 4d). The density of hair follicles was 2.7-fold greater by one-way ANOVA with Dunnett’s MCT, *p* < 0.01) in the presence of hBMP-2 with the MSC treatment. Finally, MSCs alone and MSCs + hBMP-2 significantly increased the number of blood vessels (Fig. 4e) and thickness of the endothelial barrier (Fig. 4f).

In conclusion, addition of hBMP-2 to MSC injection appears to promote angiogenesis, which is responsible for the formation of new blood vessels necessary for the repair of radiation-induced dermatitis.

### Impact of MSCs + hBMP-2 co-treatment on angiogenesis

**Figure 5:**
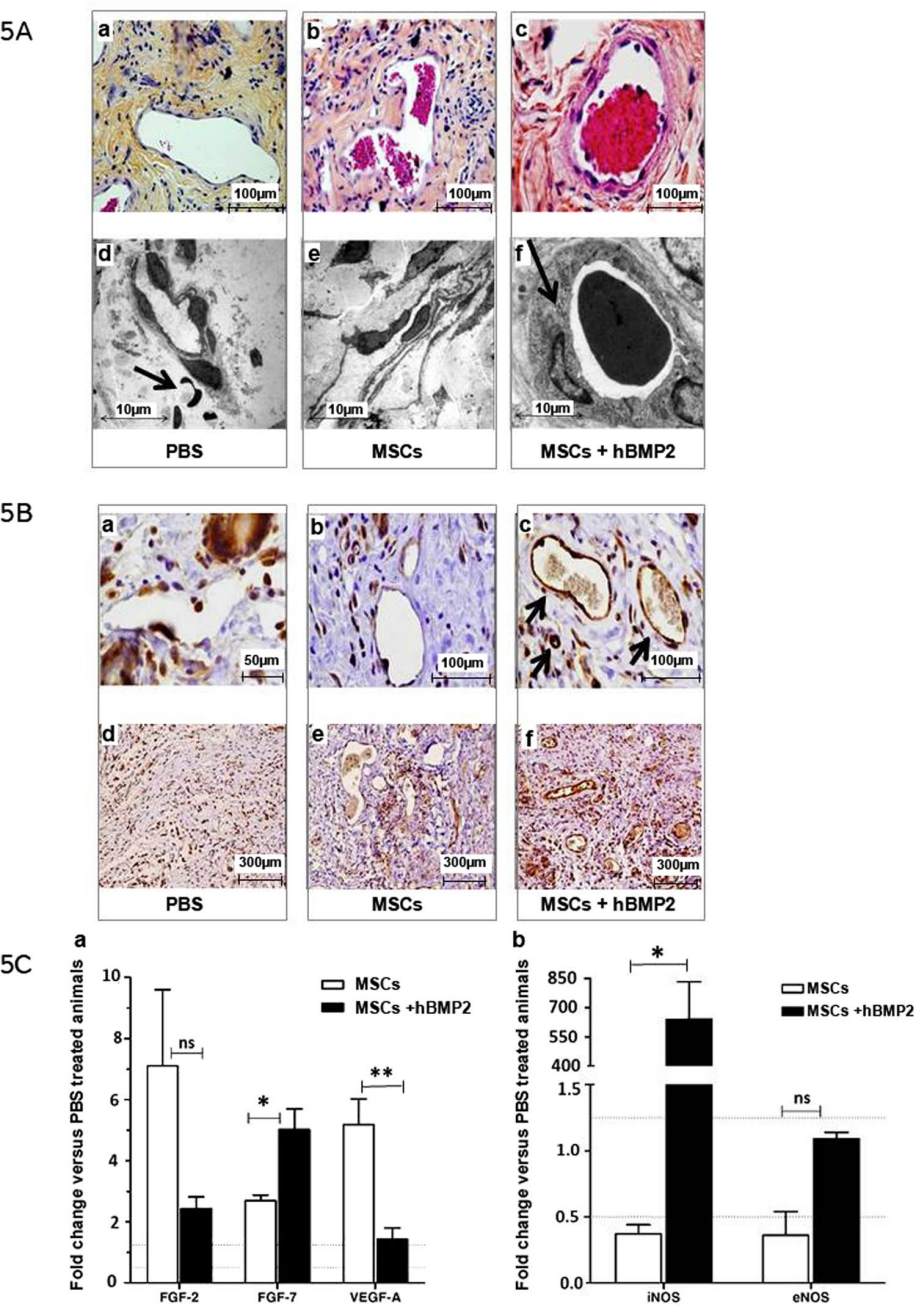
Effect of cell therapy on endothelial barrier integrity and the recruitment of CD31 + vascular cells and leukocytes one week post-treatment: (**A**) Successive histological sections (a–c) as observed under light (using HES staining) and (d–e) scanning electron microscopy. (**B**) Immunohistochemical staining of radiation-induced dermatitis sections with anti-CD31 antibody (a and d) PBS control treatment, (b and e) MSC infusion, and (c and f) MSCs + hBMP-2 co-infusion. (**C**) Relative RT-PCR quantification in MSCs (white bars) and MSCs + hBMP-2 (black bars) treated animals vs. PBS controls. (a) fibroblast growth factors (FGF-2 and FGF-7) and vascular endothelial growth factor-A (VEGF-A). (b) Inducible and endothelial nitric oxide synthases (iNos and eNos). Results were expressed as the fold change in comparison with PBS-treated animals. Each group of animals comprised 10 Sprague Dawley rats. Comparison by ANOVA followed by the Mann-Whitney or Dunnett’s test for group pair-wise comparisons. Data represent mean ± SEM, ns: not significant, *p < 0.05, **p < 0.01, ***p < 0.001.

To evaluate the degree of angiogenesis in healing tissue 1 week post-treatment, we studied the integrity of the vascular endothelial barrier using immunohistological staining and electron microscopy. As depicted in Fig. 5Aa–e, the radio-sensitive vascular endothelial barrier is highly compromised following ionizing radiation exposure. We observed heterogeneous and discontinuous distribution of endothelial cells in PBS control and MSC-injected animals (Fig. 5Aa,b,d,e). Over the same time period, restoration of a thick, homogeneous, and continuous endothelial barrier was detected following MSCs + hBMP-2 co-infusion (Fig. 5Ac,f).

We evaluated vascular integrity by staining for CD31 (platelet endothelial cell adhesion molecule-1, PECAM-1) which makes up a large portion of endothelial cell-cell junctions. Light microscopy examination of blood vessel sections immuno-stained for CD31 expression revealed a few discrete patches of endothelial cells in control PBS and MSC-treated animals (Fig. 5Ba,b). Homologous sections in animals treated with MSCs + hBMP-2 displayed a continuous layer of endothelial cells with dense cell-cell junctions on the blood vessel walls (Fig. 5Bc).

This finding suggests that MSCs + hBMP-2 co-treatment restores the integrity of the vascular endothelial barrier.

We also observed an increase in CD31 + cells in the dermis of animals treated with MSCs or MSCs + hBMP-2 (Fig. 5Be,f) compared to PBS controls (Fig. 5Bd). MSC treatment, especially with the addition of hBMP-2, hastens the remodelling phase of lesion healing by restoring the vascular system. Thus, though MSC and MSCs + hBMP-2 treatment increased the number of blood vessels and thickness of their endothelial barrier, the addition of hBMP-2 is required for recovery of the integrity and physiological functionality of the endothelial barrier. To evaluate the effect of co-injection of MSCs plus hBMP-2 on angiogenesis in healing tissue, we measured angiogenesis-related growth factors VEGF-A and factors which promote wound healing such as FGF-2 and 7. In MSC-treated rats, one week after treatment, FGF-2, FGF-7 and VEGF-A genes were up-regulated in comparison to in PBS treated animals, indicating stimulation of angiogenesis (Fig. 5Ca). However, rats treated with MSCs + hBMP-2 exhibited no or weak differential expression of angiogenesis factors compared to PBS-treated animals (FGF-2, VEGF-A) and an increase in FGF-7, as determined by a Mann-Whitney test (data representing mean ± SEM, ns: not significant, *p < 0.05, **p < 0.01, ***p < 0.001). This finding supports the fact that these two treatment groups are at different stages of angiogenesis and the repair process.

To investigate whether MSCs + hBMP-2 rats are more advanced in wound healing we measured Nitric Oxide Synthase (NO), as the different phases of the wound healing process are regulated by signalling factors, including FGF and NO. We measured inducible nitric oxide synthase (iNos) which is an inducible enzyme, and endothelial nitric oxide synthase (eNos) which is a constitutive enzyme. They are present in endothelial cells. We quantified iNos and eNos expression, and the results were given as the fold change compared to PBS-treated animals. Differential expression was measured only for iNos in MSCs + hBMP-2-treated animals in comparison with the MSCs group (Fig. 5Cb) as determined by a Mann-Whitney test (data representing mean ± SEM, ns: not significant, *p < 0.05, **p < 0.01, ***p < 0.001). 7 days after treatment, iNos is induced by pro-inflammatory cytokines and correlates with the inflammatory state observed in this treatment group (Fig. 5Bd–f). PBS-treated animals had not yet begun the healing process and were still undergoing hypoxia; MSC-treated animals were in the early stage of angiogenesis, which was still being stimulated; and MSCs + hBMP-2-treated animals were in the final tissue remodelling phase with a high level of immunological cell involvement. We hypothesized that this more rapid healing in MSCs + hBMP-2-treated animals is due to early up regulation of pro-inflammatory and down regulation of anti-inflammatory cytokines (IFN-γ, DKK1 and IL-10). On day 7 after infusion, IFN-γ, DKK1 and IL-10 levels were measured using ELISA kits in the wounded area. Nevertheless, no significant difference was observed (data not shown) in the MSC-treated group compared to the MSCs + hBMP-2-treated animals, (n = 3–4).

These results indicate that MSCs + hBMP-2 accelerate vascular network regeneration.

### Role of hBMP-2 in vasculogenesis

**Figure 6:**
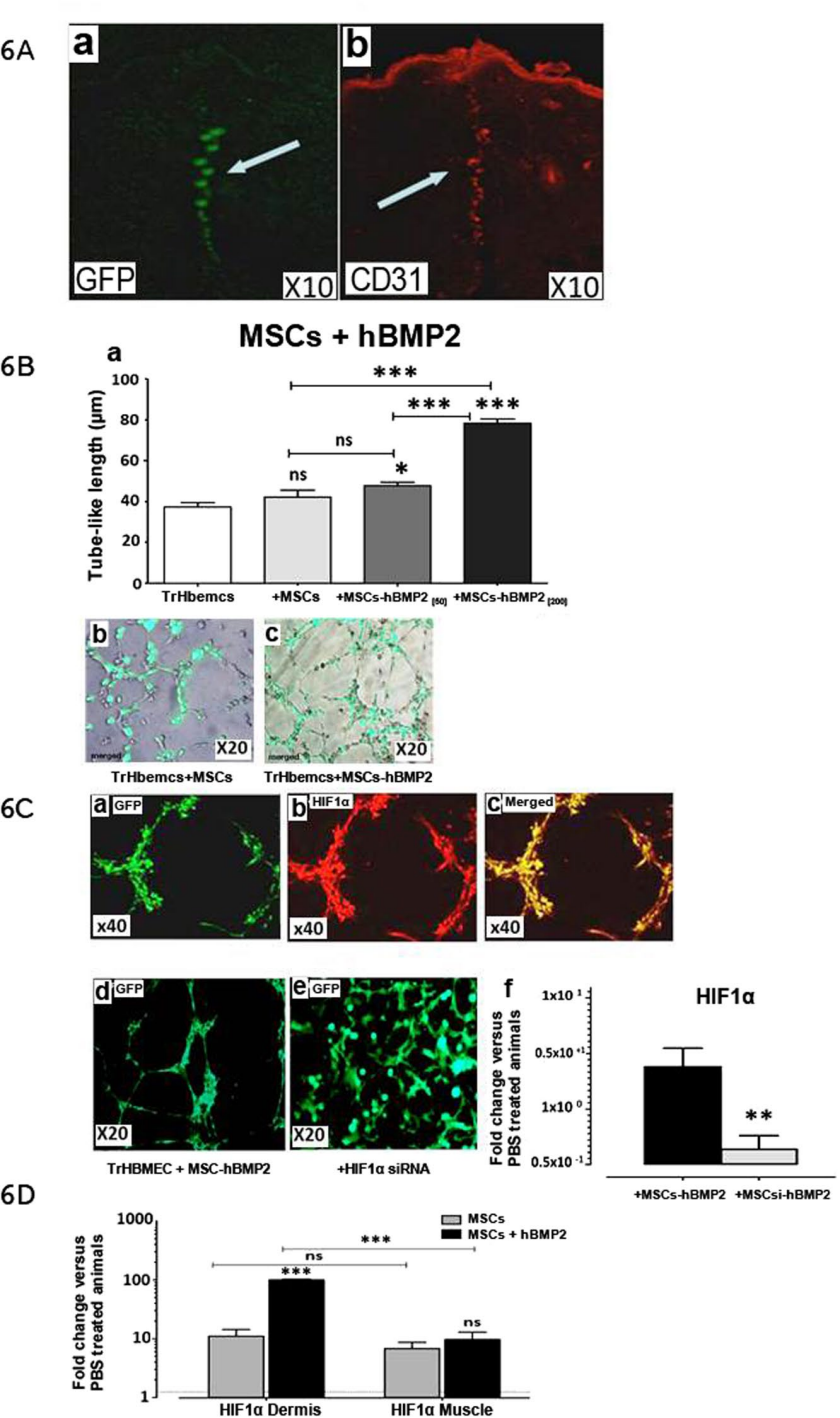
Impact of hBMP-2 addition to MSC therapy on endothelial cell recruitment in vasculogenesis. (**A**) (a and b) Co-localization of MSCs with CD31 + cells at the site of infusion. (**B**) (a) Tube length formation, human-endothelial cells (TrHBMEC, white bar) alone and with human MSCs (grey bar), and with the addition of 50 (dark grey bar) or 200 ng/ml hBMP-2 (black bar). (**C**) Fluorescent microscopy examination of the Matrigel basement membrane matrix from an *in vitro* tube formation assay with (a–d) endothelial cells (stained with alexa-coupled phalloidin), human-MSCs and hBMP-2 (TrHBMEC + MSCs + hBMP-2), staining with HIF-1 α (b), merged (c), and (e) plus siRNA in MSCs (MSCsi). (f) qRT-PCR measurement of HIF-1α RNA with siRNA (black histogram) or without white histogram. D qRT-PCR measurement of HIF-α RNA in the dermis and muscle with MSCs (grey histogram) or with MSCs plus hBMP-2 black histogram. Results were expressed as the fold change in comparison with un-irradiated (PBS-treated animals). Each group of animals comprised 10 Sprague Dawley rats. Comparison by ANOVA followed by the Mann-Whitney or Dunnett’s test for group pair-wise comparisons. Data represent mean ± SEM, ns: not significant, *p < 0.05, **p < 0.01, ***p < 0.001.

*In vivo*, local infusion of MSCs + hBMP-2 into radiation-induced dermatitis lesions revealed co-localization of MSCs with CD31 + cells, suggesting recruitment of endothelial cells at the site of injection (Fig. 6Aa,b, white arrows). To evaluate the collaborative effect of hBMP-2 and MSCs on endothelial cells for the regeneration of a vascular-like network, an *in vitro* tube formation assay was conducted as a model for assessing vasculogenesis. This assay was carried out using Matrigel and co-cultures of transformed human bone marrow endothelial cells (TrHBMECs) and human MSCs with and without the addition of hBMP-2 (Fig. 6Ba). The generation of tube-like structures by endothelial cells was more proficient in the presence of MSCs (Fig. 6Ba,b), than with the addition of hBMP-2 (Fig. 6Ba,c), which also increased the number of interconnections across tubules, forming a network. HBMP-2 also increased the length of these structures in a dose-dependent manner (Fig. 6Ba). Thus, the addition of hBMP-2 at a high dose (200 ng/ml) enhanced the rate of tube-like cell organization by endothelial cells plus MSCs and increased the length of the structures (Fig. 6Bc). MSCs provide factors such as VEGF-A, FGF, SDF-1, MMP, IGF1, vWF, GATA4 and VE-cadherin, which promote the formation of tubules in the absence of exogenous pro-angiogenic factors^30^. As the formation of tube-like structures was more efficacious in the presence of hBMP-2, this bioactive molecule is very likely involved in stimulating the production of MSC-derived pro-angiogenic factors.

One of the candidate MSC-derived pro-angiogenic factors we investigated was HIF-1α. HIF-1α, when stabilized by hypoxic conditions, up-regulates several genes, including the VEGF^31^. We investigated whether HIF-1α is required for tube formation (Fig. 6Ca–c). We studied the effect of HIF-1α knockdown by siRNA in MSCs (MSCsi) (Fig. 6Ce). Under-expression of HIF-1α using siRNA decreased endothelial tube formation and hindered the effect of hBMP-2, indicating that HIF-1α is required for the induction of MSCs + hBMP-2-mediated vascular development (Fig. 6Ce). The effect of HIF-1α knockdown by siRNA in MSCs in the presence of hBMP-2 was verified by qRT-PCR against 18 s rRNA. SiRNA decreased HIF-1α mRNA levels in comparison with the PBS-treated animals, validating the construct (Fig. 6Cf).

HIF-1α expression was measured in the dermis and muscle of rats injected with MSCs alone or MSCs + hBMP-2 (Fig. 6D) in comparison with the PBS-treated animals, one week after treatment. The expression of HIF-1α was up-regulated with both treatments. Expression of HIF-1α in the dermis was significantly increased with MSCs + hBMP-2 treatment compared to MSCs alone.

This observation reveals a tissue-specific effect from hBMP-2 and HIF-1α in the dermis.

## Discussion

MSC engraftment appears to be an interesting cell therapy option and there is clearly a need to boost its neo-vascularisation potential. This can result from the use of higher doses, repeated injections or combinations using co infusion of cytokines. We chose to use HBMP-2 which is known to play an important role in chemotaxis of microvascular endothelial cells. It is currently being used in clinical practice to enhance angiogenesis. We explored whether hBMP-2 increases the effect of MSCs on wound healing after severe irradiation-induced ulceration. We chose an external dose of 55 Gy, which induced reproducible lesions. Irradiation of the limb of the rats partially included the femur and tibia, nevertheless the percentage of irradiated bone marrow is not sufficient to induce impairment of haematopoiesis^32^.

We tested the hypothesis that hBMP-2 and MSCs have a collaborative effect on endothelial vessel formation. Our results revealed a synergistic effect of hBMP-2 and MSCs injection on the healing of radiation-induced dermatitis. HBMP-2 induced HIF-1α expression, which we thought could impact endothelial tube formation improving tissue regeneration.

Rat skin has a particular spontaneous healing potential^19^, but alopecia and the persistence of the ulcerated area remained evident at the time of sacrifice, and histological data confirmed the absence of epidermal restoration in the control group. In contrast, MSC injection improved skin healing significantly from what was described previously for mice, rats, and pigs^18,33^. In agreement with previous results, infusion of MSCs with hBMP-2 improved this beneficial effect and allowed hair regeneration^34^. The role of hBMP-2 signalling in macro-environmental hair follicle regulation was substantiated recently by other investigators^35^.

As endothelial dysfunction is one of the key mechanisms in radiation-induced dermatitis^36^, we focused on microcirculation analysis. Microvascular damage in radio-induced lesions promotes inflammatory cytokine growth factors, release of angiogenic factor, endothelial damage factors, leaky vessels, oedema, and vasoconstriction factor(s)^37^. Furthermore irradiation damage induces coagulation activation, contributing to oxygen tissue deprivation and fibrosis^38^. In irradiated tissue, the increase in vascular permeability is responsible for oedema with collagen deposits which induced fibrosis^38^. In contrast, MSCs with hBMP-2 induced increased vessel formation in the dermis than what was observed in control rats. CD31 expression was primarily absent in the controls, indicating impairment of the ultramicroscopic structure of the endothelial layer. Hair follicles were regenerated in the vicinity of these microvessels, though they were absent in the area without microvessels. Our data indicate a significant effect from MSCs with hBMP-2 on endothelial repair that may explain overall skin healing.

We tested the hypothesis that hBMP-2 and MSCs have a collaborative effect on endothelial vessel formation. We performed an *in vitro* study using an endothelial tube formation model, and we confirmed that hBMP-2 induced tube formation with Matrigel containing low doses of angiogenic factor. MSCs + hBMP-2 co-infusion induced dose-dependent tube formation with a greater effect than MSC alone. Radiation augments vasculogenesis through HIF-1-dependent SDF-1 induction^39^. HIF-1α and HIF-2α play collaborative roles in the formation of the vascular tree^40^. We observed that HIF-1α expression was enhanced in rats injected with hBMP-2. The increase in HIF-1α expression may explain faster regeneration. This result is in keeping with the observation that the loss of HIF-1α delays wound healing^28^. We demonstrated *in vitro* that hBMP-2 induces HIF-1α expression in MSCs in culture, and silencing of the HIF-1α gene inhibited the effect of hBMP-2 on endothelial tube formation. Enhancement of HIF-1α activity by non-hypoxic factors was demonstrated previously^41^. HIF-1α increases angiogenesis^30^ and might be responsible for faster wound healing in rats treated with MSCs + hBMP-2. Finally, down-regulation of the VEGF and eNos in MSCs + rhBMP-2-treated animals could be explained by more advanced in vascular network regeneration.

We conclude that hBMP-2 potentiates the effect of MSCs on minimising ulceration and improving wound healing during radiation-induced dermatitis. The combination of hBMP-2 and MSCs enhances angiogenesis and microvascular development, improving skin repair through a HIF-1-dependant mechanism. These results offer a new strategy for treating radiation-induced dermatitis, burns, and chronic ulcers. Most of all, they also suggest that addition of cytokines to MSCs may enhance their therapeutic effect in other situations where cell therapy is an option.

## Material and Methods

### Animal Model

All experiments and procedures were carried out in accordance with the Guide for the Care and Use of Laboratory Animals as published by the US National Institute of Health (NIH Publications No. 85-23, revised 1996), with European Directive (86/609/EEC), and were approved by the local ethics committee (P09-11). Six-week-old male pathogen-free Sprague Dawley rats (200–220 g, n = 140) were purchased from Charles River (L’Abresle, France) and were allocated to 14 groups (n = 10 rats in each group). The first group consisted of un-irradiated rats. Five groups were irradiated at 25, 35, 45, 55, 65, and 75 Gray (Gy). Five groups were irradiated at 55 Gy, and received 2 × 10^4^, 2 × 10^5^, 2 × 10^6^, and 2 × 10^7^ MSCs per rat, 3 weeks after irradiation. Three groups irradiated at 55 Gy were used to study the effects of co-infusion of MSCs with hBMP-2 (PBS, MSCs, MSCs + hBMP-2 groups). Irradiation was performed using an external cobalt-^60^ source emitting gamma radiation at 2 Gy/min with a collimated beam of 2 X 2 cm^2^. Selective irradiation of the right limbs (internal face) was achieved after the rats were anesthetized with isoflurane (Aerane®, Baxter, France).

### Mesenchymal Stromal Cell Isolation, Culture, and Injection

Bone Marrow (BM) cells were isolated from green fluorescence protein (GFP) transgenic Sprague-Dawley rats via femoral bone aspiration as previously described^17^ and expanded until the first passage. At passage one, cells were detached at 80% confluence and added to 2 ml of PBS supplemented with 1% bovine serum albumin (BSA, Sigma) with or without 200 ng hBMP-2 (R&D Systems, USA). An external grid was used to homogenously distribute injections. The first injections were performed at the edge of the lesion, and the subsequent injections at the centre of the ulceration. MSCs were injected at 18 points to cover a surface area of 2 cm^2^ in muscle and skin. In order to determine the optimal dose of MSCs injected, a dose range of 2 × 10^5^ to 2 × 10^7^ cells was tested per rat. MSC injection alone (n = 10) was compared to co-infusion of hBMP-2 and MSCs (n = 10, MSCs + hBMP-2 group). The control group was irradiated and injected with the vehicle (PBS + BSA). This control group was called PBS-treated animals.

Clinical Observations Sequential observations were made to estimate the ulceration surface area qualitatively and quantitatively. A previously reported phenotypic scale adapted from LENT-SOMA and Douglas-Fowler scales^42,43^ was used. The phenotypic score of the skin lesion was 0 for normal, 1 for dry desquamation, 2–3 for moist desquamation and 4 for ulceration. The surface area of the ulcerated region was quantified using a specific algorithm (Optimas, Imasys, France).

### Histological study

Histological studies were performed as previously described^17^. Angiogenesis was quantified using four fields of view for each rat for the area of skin healing with x20 magnification. The images were processed using an algorithm (Optimas, Imasys, France) that enabled the identification and numbering of the circular edges.

### Confocal Microscopy

The images were recorded on a FV500 confocal microscope (Olympus®) using a FluoView500 algorithm. Frozen tissue was cut into thin 12-µm slices and exposed to primary antibodies (goat polyclonal anti rat CD31 sc-1506, Santa Cruz Biotechnology, Santa Cruz, CA, USA) at 4 °C for 12 h. The antigen–antibody reaction was detected using a molecular probe Alexa Fluor dye-specific secondary antibody (rabbit anti-goat alexa fluor 568, Interchim, France) and the positive reaction was visualized by fluorescent microscopy without mounting medium.

### Electron Microscopy

Tissues were fixed in 1% glutaraldehyde and 4% paraformaldehyde in 0.1 M phosphate buffer (pH 7.4), post-fixed in 2% osmium tetroxide, dehydrated in graded ethanol, and embedded in Epon. Ultrathin sections cut on a Reichert Ultracut E were contrasted with uranyl acetate and lead citrate and observed using a JEOL 1010 transmission electron microscope at an accelerating voltage of 80 kV.

Detection and Quantitative Analysis of Engrafted GFP-MSC DNA extraction and PCR analysis were performed as previously described using GFP primers^17^.

### ELISA assays

Interferon-c (IFN-c), Dickkopffactor-1 (DKK-1), and IL-10 were detected using ELISA kits (R&D system) according to the manufacturer’s instructions.

*In Vitro* Endothelial Cell Capillary Tube Formation Transformed human bone marrow endothelial cells (TrHBMECs) were seeded on Matrigel, growth factor-reduced (BD Biosciences, USA), at a density of 10^4^ cells per cm^2^ and expanded for 24 h in serum containing 50% free endothelial cell basal medium and 50% αMEM (n = 3). Endothelial tube-like formation was evaluated at 6 h. This experiment was repeated with or without human MSCs and with or without hBMP-2 (50 or 200 ng/ml). HIF-1a was stained with anti-HIF-1 alpha antibody (ab8366, Origene) and revealed by a secondary Goat Anti-Mouse IgG H&L (Alexa Fluor® 647) antibody (ab150115, Origene). To test the specific role of HIF-1α, siRNA (5 nM, incubation for 6 h) was used according to the supplier’s recommendations (Qiagen). Capillary tubes were stained with alexa-coupled phalloidin (Alexa Fluor® 488 Phalloidin, A12379, Thermofisher Scientific).

The development of capillary tubes was evaluated using specific software (Optimas, Imasys, France). The perimeter outlined by tube formations observed on ten fields of view was calculated.

siRNA Knockdown by Reverse Transfection siRNAs for HIF-1α (SI00436324, Qiagen), negative control (SI03650318, Qiagen), and Hiperfect Reagent (301704, Qiagen), were obtained from Qiagen and used according to the manufacturer’s protocol for adherent cells.

### Statistical Analysis

Values were expressed as mean ± standard error of the mean (SEM) and compared using ANOVA, followed by the Mann-Whitney or Dunnett’s test for group pair-wise comparisons with significance set at *p* < 0.05.

### Ethical Approval

All experiments and procedures were carried out in accordance with the Guide for the Care and Use of Laboratory Animals as published by the US National Institute of Health (NIH Publications No. 85–23, revised 1996), European Directive (86/609/EEC), and were approved by the local ethics committee (P09-11). The Institute for Radiological Protection and Nuclear Safety (IRSN) approved the experimental protocols.
