# Author Correction: Synergistic effect of human Bone Morphogenic Protein-2 and Mesenchymal Stromal Cells on chronic wounds through hypoxia-inducible factor-1 α induction

**DOI:** 10.1038/s41598-018-23594-x

**Published:** 2018-04-11

**Authors:** Sabine François, Véronique Eder, Karim Belmokhtar, Marie-Christine Machet, Luc Douay, Norbert-Claude Gorin, Marc Benderitter, Alain Chapel

**Affiliations:** 1 Laboratory of Research on Irradiated Healthy Tissue Regeneration (LR2I), Institute for Radiological Protection and Nuclear Safety (IRSN), F-92260 Fontenay-aux-Roses, France; 2 Proliferation and Differentiation of Stem Cells, Centre de Recherche Saint-Antoine (CRSA), UMR_S938, Faculté de médecine Pierre et Marie Curie, France Institut National de la Santé et de la Recherche Médicale (INSERM) U938, 27 rue de Chaligny, 75012 Paris, Paris France; 3 LAB.P.ART.-EA3852 Faculty of Medicine, University of Tours, 2 bis boulevard Tonnellé, 37000 Tours, France

Correction to: *Scientific Reports* 10.1038/s41598-017-04496-w, published online 27 June 2017

The original version of this Article contained a typographical error in the spelling of the authors Alain Chapel and Véronique Eder, which were incorrectly given as Chapel A. Alain and Véronique V. Eder. This has now been corrected in the PDF and HTML versions of the Article.

